# Prevalence of chronic and multisite pain in adolescents and young adults with ADHD: a comparative study between clinical and general population samples (the HUNT study)

**DOI:** 10.1007/s00787-023-02249-x

**Published:** 2023-06-29

**Authors:** Ingunn Mundal, Jorun Schei, Stian Lydersen, Per Hove Thomsen, Torunn Stene Nøvik, Levi R. Kvitland

**Affiliations:** 1 Regional Centre for Child and Youth Mental Health and Child Welfare (RKBU), Department of Mental Health, Faculty of Medicine and Health Sciences, Norwegian University of Science and Technology (NTNU), Trondheim, Norway; 2 Department of Health and Social Sciences, Molde University College, Molde, Norway; 3 Kristiansund Community Mental Health Centre, Division of Psychiatry, Møre and Romsdal Hospital Trust, Kristiansund, Norway; 4 Department of Child and Adolescent Psychiatry, Aarhus University Hospital, Psychiatry, Aarhus N, Denmark; 5 St. Olavs University Hospital, Trondheim, Norway

**Keywords:** ADHD, Adolescents, Chronic pain, Multisite pain, Single-site pain, HUNT

## Abstract

Attention-deficit/hyperactivity disorder (ADHD) and chronic pain are prevalent and associated. We examined the prevalence and distribution of chronic pain in adolescents and young adults with ADHD using 9-years longitudinal data (from T1:2009–2011 to T3:2018–2019) with three time points from a clinical health survey compared to two age-matched reference population-based samples. Mixed-effect logistic regression and binary linear regression were used to estimate the probability for chronic and multisite pain at each time point and to compare the prevalence of chronic pain with the reference populations. The prevalence of chronic and multisite pain was high in those with ADHD, especially in female young adults, with highly prevalent chronic pain at 9 years of follow-up (75.9%) compared to 45.7% in females in the reference population. The probability of having pain was only statistically significant for chronic pain in males at 3 years of follow-up (41.9%, *p* = 0.021). Those with ADHD were at higher risk of reporting single-site and multisite pain compared to the general population at all measurement points. Longitudinal studies should be tailored to further understand the complex sex differences of comorbid chronic pain and ADHD in adolescents, exploring predictive factors of pain assessing long-term associations with bodyweight, psychiatric comorbidities, and possible mechanisms of stimulant use effects on pain.

## Introduction

Attention-deficit/hyperactivity disorder (ADHD) typically emerges in childhood with an average global prevalence of 5%, often with difficulties continuing into adulthood [1], frequently showing high concurrent comorbidity with other neurodevelopmental and psychiatric disorders [2]. ADHD diagnosis differs in level of core symptoms (inattentive or hyperactive presentation, or both), impairments, and comorbidities [3]. Comorbidities are significantly associated with somatic complaints such as pain [2, 4, 5], and persistent impairment in adult life [6]. Chronic pain, which typically is defined as pain lasting more than 3 months [7], is commonly reported by adolescents, with a wide variability in the reported prevalence estimates, varying according to age, sex (more prevalent in females), and country of origin [8, 9]. Its recurrent nature indicates that having chronic pain in adolescence is highly predictive of chronic pain in adulthood [10]. A recent systematic review examining prognostic factors of adolescent chronic pain found that chronic pain persisted in more than 50% of the adolescents at 9-year follow-up, with persistence associated with pain in seven out of nine single pain sites, most frequently occurring in the lower back, neck, and knee [11]. Female sex was commonly associated with pain at follow-up, with pain across different pain sites such as headache, pain in the upper extremities, lower extremities, chest, and back [11]. In adults, the number of pain sites is associated with poorer prognosis compared to single-site pain [12]; however, over time, studies of adult populations have shown relatively stable trajectories of pain [13].

In community prevalence studies of adolescents, ADHD was found to be twice or three times more common among males than females [1], suggesting an under-recognition of ADHD in females [14]. The clinical presentation, pharmacological treatments, and course of ADHD have been extensively characterized; however, without addressing the broader, long-term clinical needs [15]. For example, chronic pain shares common mechanisms and concerns with ADHD; pain has well-established effects on attention and vice versa [5, 16]. However, there is little detailed knowledge regarding somatic diseases in those with ADHD from a longitudinal perspective, and the extent to which ADHD alone is related to somatic complaints such as chronic pain.

Somatic comorbidity addressing the prospective course of chronic pain from adolescents to young adulthood in ADHD has received less attention in the research literature. This study aimed to (1) report the prevalence of chronic pain in adolescents and young adults with ADHD using longitudinal data from a clinical health survey and (2) compare the prevalence of chronic pain in those with ADHD using two age-matched reference population samples.

## Methods

### Sample and procedures

**Fig. 1:**
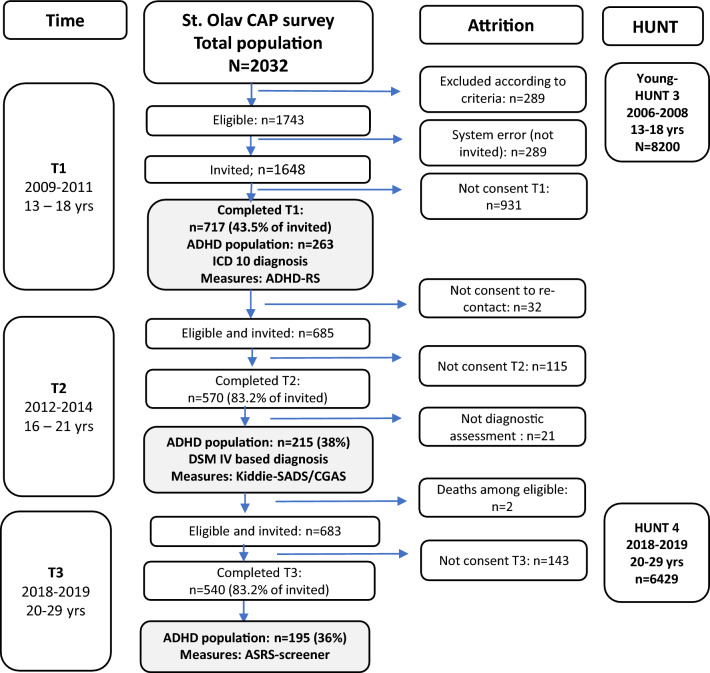
Flowchart of inclusion and attrition

Data were obtained from a health survey in the Department of Children and Youth Division of Mental Health Care at St. Olav’s University Hospital, Trondheim University Hospital, Norway (the CAP study), along with a large Norwegian cohort of adolescents and young adults in the Norwegian county of Trøndelag (The Young-HUNT and HUNT surveys). Figure 1 presents the participant flow in these studies.

Of the total of 717 participants in the CAP study at baseline (T1), *n* = 263 had ADHD diagnosis, and were included in this study. The mean (SD) age was 15.7 (1.65) years, ranging from 13.0 to 20.5, and 45% were females (*n* = 106). Adolescents enrolled at the CAP clinic received verbal and written invitations during their first visit after the project started. For participants younger than 16 years of age, parental consent was obtained; participants aged ≥ 16 years provided written informed consent to participate. From the CAP study, only those with ADHD were included.

Data from the reference population-based Young-HUNT3 study (the adolescent part of the HUNT [the Trøndelag Health Study] were collected from the third wave of the Young-HUNT study and the fourth wave of the HUNT study (HUNT4), which were conducted from 2006 to 2008 and 2018–2019, respectively, and corresponded to times T1 and T3 in the CAP study. Of the 10,464 adolescents aged 13–19 years in Trøndelag County of Norway who were invited to participate, 8200 (78.4%) participated [17]. The Young-HUNT studies aimed to capture rapidly changing health statuses, behaviors, and functions that differed from those of adults; data collection included self-reported questionnaires, and clinical measurements [18]. To ensure maximum comparability, health data in the HUNT studies were collected comparably using interviews, identical self-administrated questionnaires, including screening for ADHD problems (adolescent’s school functioning in Young-HUNT3 [19] and Adult ADHD self-report scale in HUNT4 [20]), and clinical examinations, which corresponded to the three waves of the CAP study. During the school day, students completed the questionnaires printed with a unique barcode without names or other identifiers; questionnaires were sealed in a blank envelope by the student after completion. We included subjects from the HUNT4-study from the same age group (20–29 years old) as participants in CAP-T3.

### Measures

Clinical medical and psychiatric diagnostic assessments followed standardized diagnostic processes and routine assessments according to the ICD-10 [21] and the Norwegian national guidelines for ADHD [22, 23]. The procedures included information from patients and their parents and teachers regarding developmental history, somatic health status, and school functioning [24, 25]. Assessments of emotional and behavioral problems were obtained from the Achenbach System of Empirically Based Assessment (ASEBA) checklists [26] and ADHD symptoms from the ADHD Rating Scale-IV parent report (ADHD-RS-IV) [27].

Chronic pain was defined as having pain in at least one of the musculoskeletal locations occurring at least once a week in the last 3 months, and multisite pain was defined as having chronic pain in three or more locations [7, 28]. The self-reported pain questionnaires in the CAP-T1 and T3 surveys included questions regarding somatic pain symptoms that had been experienced during the last 3 months and were not related to any known disease or injury [29]. Questions from Mikkelsson et al. were used to determine the duration and location of pain, which was reported to have good test–retest reliability in detecting those who had pains at least once a week (Cohen’s kappa was 0.90). Observed agreement between pain questionnaire and interview technique was 86% and kappa was 0.67 [29]. The adolescents were asked to specify pain in different locations such as headache, abdomen, chest, upper or lower back/buttocks/limbs with five response alternatives including the frequency of experiencing pain, specified as “never/seldom”, “once a month”, “once a week”, “more than once a week” or “almost every day” [29, 30]. Assessment of pain locations was assisted by a pain mannequin and was considered appropriate to be completed unassisted by children older than 8 years of age [31]. The main outcome variable (chronic pain) was analyzed as categorical, with categories of 0 (no pain) and 1 (pain).

### Statistical analyses

For those with ADHD in the CAP study, we used mixed-effect logistic regression with dichotomized chronic and multisite pain, one at a time, as dependent variables, time point (T1, T2, and T3) as a categorical covariate, and participant as a random effect. Participants without data at all time points were also included in the mixed-effect logistic regression model, as such results are approximately unbiased when data are missing at random [32]. We have presented the estimated prevalence rates (probabilities) calculated from the estimated odds from these analyses as *p* = odds/(odds + 1).

To compare the prevalence of chronic pain in adolescents with ADHD in CAP T1 and young adults in T3 with those in Young-HUNT3 and HUNT4, we performed binary linear regression analyses with pain as dependent variable and CAP versus HUNT, age, and sex as covariates. This analysis was performed separately for each of the site-specific pain variables, chronic pain, and multisite pain, and with separate analyses for males and females. We have reported risk difference (RD) with 95% confidence intervals (CI). The level of significance was set to *p* < 0.05. All data were analyzed using Stata 17 for Windows (Stata Corporation, USA).

### Ethics

Written informed consent from adolescents and their parents was obtained prior to inclusion in both the CAP study and the Young-HUNT study. Study approval was granted by the Regional Committee for Medical and Health Research Ethics (reference 4.2008.1393 number for the CAP study, 4.2006.250 for the Young-HUNT3 study, 2011//2061/REK-Midt and for the present study).

## Results

### Sample characteristics and chronic pain prevalence

Table 1 shows the characteristics of both populations. From the Young-HUNT3 study (N = 8312), the mean age was 15.9 (1.75) years, ranging from 12.7 to 20.9, and 50.3% (*n* = 4181) were females. The mean number of pain sites was higher in CAP participants as compared to the general population. In the CAP study, the prevalence of multisite pain in females had decreased at 9-year follow-up but had increased in males. In the general population, the prevalence of multisite pain in both sexes had increased from Young-HUNT3 to HUNT4 but was still lower than that observed for in CAP. Additional descriptive statistics are published elsewhere [2].

**Table 1:** Sample characteristics

	T1 (CAP) *n* = 263 (2009–2011)		T2 (CAP) *n* = 215 (2012–2014)		T3 (CAP) *n* = 195 (2018–2019)		Young-HUNT3 *n* = 8200 (2006–2008)		HUNT4 *n* = 6428 (2018–2019)	
	Females	Males	Females	Males	Females	Males	Females	Males	Females	Males
Sex % (*n*)	40.3 (106/263)	59.7 (157/263)	41.4 (89/215)	58.6 (126/215)	45.6 (89/195)	54.4 (106/195)	50.3 (4128/8200)	59.7 (4072/8200)	56.5 (3633/6428)	43.5 (2795/6428)
Age (SD)	15.8 (1.89)	15.2 (1.50)	18.9 (1.87)	18.1 (1.48)	24.6 (1.82)	24.2 (1.45)	15.9 (1.76)	15.9 (1.72)	24.8 (3.01)	24.7 (3.01)
Chronic pain % (*n*)	81.0 (81/100)	56.6 (82/145)	78.8 (67/85)	44.0 (51/116)	75.9 (66/87)	51.9 (54/104)	51.1 (2000/3195)	31.4 (1191/3778)	45.7 (1510/3303)	25.0 (642/2565)
Multisite pain % (*n*)	49.0 (49/100)	19.3 (28/145)	51.3 (159)	16.5 (389)	42.5 (37/87)	23.1 (23/104)	20.1 (781/3883)	9.1 (343/3757)	26.3 (632/2408)	16.1 (224/1389)
Sum pain sites mean (SD)	2.8 (2.3)	1.3 (1.7)	3.4 (3.2)	1.2 (2.0)	3.2 (2.6)	1.6 (2.6)	1.3 (1.7)	0.7 (1.3)	1.9 (1.8)	1.2 (1.8)

Those with ADHD included HUNT4: those younger than 30 years included

*CAP* child and adolescent psychiatry (the CAP study)

### The course of chronic and multisite pain in those with ADHD

Table 2 shows the estimated probability of having chronic pain and multisite pain at each time point in those with ADHD in the CAP study. The probability of having chronic pain at baseline for the total sample was 74.3% with chronic pain decreasing at T2 (62.3%, *p* = 0.027), and increasing at T3 (66.4%, *p* = 0.14). For multisite pain at baseline, the probability was 22.9%, with decreasing probability at T3 (21.5%, *p* = 0.76).

**Table 2:** Estimated chronic pain prevalence in those with ADHD

	Total			Females			Males		
	Probability (%)	95% CI (%)	*p* value	Probability (%)	95% CI (%)	*p* value	Probability (%)	95% CI (%)	*p* value
Chronic pain									
Baseline T1 2009–2011	74.3%	65.4–81.6		90.4%	78.9–96.0		59.2%	48.0–69.2	
Follow-up T2 2012–2014	62.3%	51.9–71.7	**0.027**	87.9%	74.7–94.7	0.57	41.9%	30.7–53.9	**0.021**
Follow-up T3 2018–2019	66.4%	56.0–75.4	0.14	85.4%	71.4–93.2	0.29	50.5%	38.1–62.9	0.26
Multisite pain									
Baseline T1 2009–2011	22.9%	15.9–31.8		48.9%	36.2–61.7		8.9%	4.0–18.6	
Follow-up T2 2012–2014	25.4%	17.4–35.6	0.60	55.3%	41.3–68.5	0.46	8.8%	3.7–19.4	0.96
Follow-up T3 2018–2019	21.5%	14.2–31.3	0.76	39.4%	26.8–53.4	0.27	11.6%	5.2–23.7	0.48

The level of significance was set to *p* < 0.05 (in bold)

Based on mixed-effect logistic regression with pain as dependent variable and time as categorical covariate. *p* values for change from baseline

For females, probability for chronic pain at baseline was 90.4% but decreased at T3 (85.4%, *p* = 0.27). For multisite pain, the baseline probability was 48.9% with a decrease at T3 (39.4%, *p* = 0.76).

For males, the probability of chronic pain at baseline was 59.2%, but decreased significantly at T2 (41.9%, *p* = 0.021), and increased at T3 (50.5%, *p* = 0.26). For multisite pain, the probability was 8.9% (T1) and 11.6%, *p* = 0.48 (T3), respectively.

### Pain sites prevalence in those with ADHD compared to the general population

Table 3 presents the estimated prevalence of chronic pain separately for each sex at each time point in CAP and the general population. The frequency of chronic pain was higher in adolescents and young adults with ADHD than in those in the reference populations, at all measurement points, but decreased with time. Table 3 presents risk differences for pain between adolescents with ADHD in CAP-T1 compared to those in Young-HUNT3, estimated from the binary linear regression and adjusted for age and sex. The risk differences were significantly higher in those with ADHD considering headache (RD = 12.2%, CI 0.07–0.18), and pain in various locations, with highest difference in neck (RD = 16.9%, CI 0.11–0.23), lower back (RD = 13.7%, CI 0.08–0.19), upper back (RD = 12.1%, CI 7.0–17.1), stomach (RD = 13.5%, CI 0.09–0.18), and other sites (RD = 26.0%, CI 0.20–0.32), as well as chronic pain (RD = 25.1%, CI 0.20–0.31) and multisite pain (RD = 17.1%, CI 0.12–0.23), compared to the general population.

**Table 3:** Pain prevalence in CAP-T1 and Young-HUNT3

Pain locations	T1 (*n* = 263)	YH3 (*n* = 8312)	Difference % 95% CI	*p *value
Head	33.3% (85/255)	22.4% (1896/8033)	**12.2%** [6.7–17.8%]	** < 0.001**
Neck	33.9% (86/254)	17.8% (1418/7969)	**16.9%** [11.3–22.5%]	** < 0.001**
Upper back	21.5% (54/251)	10.0% (802/7994)	**12.1%** [7.0–17.1%]	** < 0.001**
Lower back	27.8% (72/255)	14.6% (1172/8016)	**13.7%** [8.4–19.0%]	** < 0.001**
Chest	10.6% (27/254)	4.4% (352/7994)	**6.2%** [2.5–9.9%]	**0.001**
Gastrointestinal	25.1% (64/255)	11.6% (923/985)	**13.5%** [8.5–18.5%]	** < 0.001**
Left arm	7.4% (19/256)	2.7% (212/7992)	**4.7%** [1.5–7.9%]	**0.004**
Right arm	6.3% (16/255)	3.1% (248/7988)	**2.9%** [0.0–5.9%]	**0.049**
Left leg	14.3% (37/258)	8.0% (643/8002)	**5.7%** [1.5–9.9%]	**0.008**
Right leg	15.6% (40/257)	8.9% (715/8016)	**− 1.4%** [− 1.7% to − 1.0]	** < 0.001**
Other pain	33.2% (83/250)	7.3% (543/7453)	**26.0%** [20.2–31.9%]	** < 0.001**
Chronic pain	66.5% (163/245)	44.5% (3401/7640)	**25.1%** [19.5–30.8%]	** < 0.001**
Multisite pain	31.4% (77/245)	14.7% (1124/7640)	**17.1%** [11.6–22.7%]	** < 0.001**
Females	40.3% (106/263)	50.3% (4181/8312)		
Head	49.5% (52/105)	30.4% (1237/4066)	**19.3%** [9.8–28.9%]	** < 0.001**
Neck	50.5% (53/105)	23.0% (927/4039)	**28.0%** [18.6–37.3%]	** < 0.001**
Upper back	25.7% (26/101)	12.8% (519/4047)	**12.9%** [4.4–21.4%]	**0.003**
Lower back	38.1% (40/105)	17.7% (719/4061)	**20.2%** [11.2–29.3%]	** < 0.001**
Chest	16.0% (17/106)	5.2% (211/4050)	**10.8%** [3.8–17.8%]	**0.002**
Gastrointestinal	40.6% (43/106)	17.5% (709/4053)	**23.1%** [13.9–32.4%]	** < 0.001**
Left arm	7.6% (8/106)	2.8% (115/4059)	**4.7%** [− 0.3% to 9.8%]	0.067
Right arm	6.6% (7/106)	3.3% (133/4055)	**3.1%** [− 1.5% to 7.7%]	0.19
Left leg	18.9% (20/106)	9.2% (374/4055)	**9.3%** [2.0–16.7%]	**0.013**
Right leg	22.9% (24/105)	10.0% (405/4060)	**12.9%** [4.8–21.0%]	**0.002**
Other pain	40.2% (42/102)	8.7% (325/3743)	**31.5%** [22.0–41.1%]	** < 0.001**
Chronic pain	81.0% (81/100)	54.6% (2120/3883)	**27.0%** [19.4–34.5%]	** < 0.001**
Multisite pain	49.0% (49/100)	20.1% (781/3883)	**29.2%** [19.5–38.8%]	** < 0.001**
Males	59.7% (157/263)	49.7% (4131/8312)		
Head	22.0% (33/150)	14.1% (559/3967)	**8.0%** [1.3–14.8%]	**0.019**
Neck	22.2% (33/149)	12.5% (491/3930)	**10.1%** [3.4–16.9%]	**0.003**
Upper back	18.7% (28/150)	7.2% (283/3947)	**11.5%** [5.2–17.8%]	** < 0.001**
Lower back	20.7% (31/150)	11.5% (453/3955)	**9.7%** [3.4–16.1%]	**0.003**
Chest	6.8% (10/148)	13.6% (41/3994)	**3.4%** [− 0.7% to 7.6%]	0.103
Gastrointestinal	14.1% (21/149)	5.4% (214/3932)	**8.3%** [2.7–13.9%]	**0.004**
Left arm	7.3% (11/150)	2.5% (97/3933)	**4.6%** [0.4–8.7%]	**0.031**
Right arm	6.0% (9/149)	2.9% (115/3933)	**2.8%** [− 1.0% to 6.7%]	0.15
*Left leg	11.2% (17/152)	6.8% (269/3949)	**4.4%** [− 0.7% to 9.4%]	0.091
Right leg	10.5% (16/152)	7.8% (310/3956)	**1.7%** [− 3.1% to 6.6%]	0.48
Other pain	28.4% (31/148)	5.9% (218/3710)	**22.4%** [15.1–29.7%]	** < 0.001**
Chronic pain	56.6% (82/145)	34.1% (1282/3757)	**22.3%** [14.1–30.6%]	** < 0.001**
Multisite pain	19.3% (28/145)	9.1% (343/3757)	**10.1%** [3.6–16.7%]	**0.002**

The level of significance was set to *p* < 0.05 (in bold)

Confidence intervals (CI) and *p* values from binary linear regression, adjusted for age and sex in total sample

*ADHD* attention-deficit hyperactivity disorder, *CAP* child and adolescent psychiatry, *T1* baseline (2009–2011), *YH3* the Nord-Trøndelag Health Study—Young-HUNT3 (2006–2008)

*Unadjusted for age. Computation did not converge when adjusting for age

Table 4 presents results for those with ADHD aged 20–29 years in CAP-T3 compared to those in HUNT4 in the same age range. The risk differences were significantly higher in those with ADHD, considering pain in the shoulders (RD = 18.1%, CI 12.0–24.3), neck (RD = 13.4%, CI 0.08–0.19), lower back (RD = 13.9%, CI 0.08–0.20), knee (RD = 13.4%, CI 0.08–0.19), and ankle (RD = 12.1%, CI 0.07–0.17) as well as bilateral pain (RD = 53.6%, CI 0.43–0.64) and multisite pain (RD = 11.3%, CI 0.05–0.18), but not chronic pain, compared to the general population.

**Table 4:** Pain prevalence in CAP-T3 and HUNT4

	T3 (n = 195)	HUNT4 (*n* = 6428)	Difference % 95% CI	*p* value
Head	51.8% (99/191)	50.8% (1927/3797)	**5.7%** [− 1.3% to 12.7%]	0.11
Jaw	8.2% (16/195)	2.1% (133/6428)	**6.0%** [2.4–9.7%]	**0.001**
Neck	23.6% (46/195)	10.8% (695/6428)	**13.4%** [7.7–19.1%]	** < 0.001**
Upper back	15.4% (30/195)	6.6% (422/6428)	**9.2%** [4.2–14.1%]	** < 0.001**
Lower back	24.1% (47/195)	10.3% (663/6428)	**13.9%** [8.1–19.7%]	** < 0.001**
Chest	7.7% (15/195)	1.7% (111/6428)	**5.9%** [2.3–9.6%]	**0.001**
Shoulders	27.2% (53/195)	9.4% (603/6428)	**18.1%** [12.0–24.3%]	** < 0.001**
Elbow	2.6% (5/195)	1.8% (117/6428)	**0.8%** [− 1.4% to 3.1%]	0.48
Hand	11.8% (23/195)	4.9% (314/6428)	**6.9%** [2.6–11.3%]	**0.002**
*Hip	11.3% (22/195)	4.9% (312/6428)	**6.6%** [2.2–11.1%]	**0.004**
Thigh	5.6% (11/195)	1.2% (80/6428)	**4.4%** [1.2–7.5%]	**0.007**
Knee	19.5% (38/195)	6.4% (410/6428)	**13.4%** [7.9–18.9%]	** < 0.001**
Leg	5.1% (10/195)	2.3% (149/6428)	**2.9%** [− 0.1% to 5.9%]	0.060
Ankle	15.9% (31/195)	4.1% (263/6428)	**12.1%** [7.0–17.2%]	** < 0.001**
Bilateral pain	67.6% (50/74)	15.2% (978/6428)	**53.6%** [43.4–63.7%]	** < 0.001**
Chronic pain	62.8% (120/191)	62.8% (2383/3797)	**4.1%** [− 2.7–10.9%]	0.24
Multisite pain	31.9% (61/191)	22.6% (857/3797)	**11.3%** [4.7–17.8%]	**0.001**
Females	40.3% (106/263)	56.5% (3633/6428)		
Head	66%.7 (58/87)	59.3% (1429/2408)	**7.3%** [− 2.7% to 17.5%]	0.15
Jaw	49.5% (52/105)	3.1% (111/3633)	**9.3%** [2.5–16.2%]	**0.008**
Neck	33.7% (30/89)	14.9% (541/3633)	**18.9%** [9.0–28.8%]	** < 0.001**
Upper back	20.2% (18/89)	8.4% (305/3633)	**11.9%** [3.5–20.3%]	**0.006**
Lower back	34.8% (31/89)	12.8% (464/3633)	**22.1%** [12.1–32.0%]	** < 0.001**
Chest	11.2% (10/89)	2.3% (83/3633)	**9.0%** [2.4–15.5%]	**0.008**
Shoulders	37.1% (33/89)	12.2% (443/3633)	**24.9%** [14.8–35.0%]	** < 0.001**
Elbow	2.3% (2/89)	1.8% (65/3633)	**0.4%** [− 2.6% to 3.5%]	**0.79**
Hand	18.0% (16/89)	6.3% (228/3633)	**11.7%** [3.7–19.7%]	**0.004**
Hip	19.1% (17/89)	7.2% (260/43633)	**12.2%** [3.9–20.5%]	**0.004**
Thigh	7.9% (7/89)	1.7% (60/3633)	**6.2%** [0.6–11.8%**]**	**0.030**
Knee	23.6% (21/89)	7.9% (287/3633)	**15.7%** [6.8–24.5%]	**0.001**
Leg	6.7% (6/89)	3.0% (108/3633)	**3.8%** [− 1.5% to 9.0%]	0.16
Ankle	19.1% (17/89)	5.4% (195/3633)	**13.7%** [5.5–21.9%]	**0.001**
Bilateral pain	82.9% (34/41)	19.8% (719/3633)	**62.9%** [51.2–74.6%]	** < 0.001**
Chronic pain	75.9% (33/87)	69.6% (1677/2408)	**6.2%** [− 2.9% to 15.4%]	0.18
Multisite pain	42.5% (37/87)	26.3% (633/2408)	**16.4%** [5.8–26.8%]	**0.002**
Males	54.4% (106/195)	43.5% (2795/6428)		
Head	39.4% (41/104)	35.6% (498/1389)	**4.4%** [− 5.4% to 14.1%]	0.38
Jaw	4.7% (5/106)	2.1% (22/27957)	**3.9%** [− 0.1% to 8.0%]	0.056
Neck	15.1% (16/106)	5.5% (154/2795)	**10.0%** [3.1–16.9%]	**0.005**
Upper back	11.3% (12/106)	4.2% (117/2795)	**7.4%** [1.3–13.5%]	**0.018**
Lower back	15.1% (16/106)	7.1% (199/2795)	**8.2%** [1.3–15.0%]	**0.020**
Chest	4.7% (5/106)	1.0% (28/2795)	**3.8%** [− 0.3% to 7.9%]	0.066
Shoulders	18.9% (20/106)	5.7% (160/2795)	**13.3%** [5.0–20.1%]	**0.001**
Elbow	2.8% (3/106)	1.9% (52/2795)	**1.2%** [− 2.1% to 4.5%]	0.47
Hand	6.6% (7/106)	23.1% (86/2795)	**3.9%** [− 1.0% to 8.8%]	0.12
*Hip	7.2% (5/106)	1.9% (52/2795)	**3.0%** [− 1.0% to 7.0%]	0.15
Thigh	3.8% (4/106)	0.7% (20/2795)	**3.1%** [ −0.5% to 6.7%]	0.096
Knee	16.0% (17/106)	4.4% (123/2795)	**11.8%** [4.8% to 18.8%]	**0.001**
Leg	3.8% (4/106)	1.5% (41/2795)	**2.3%** [− 1.3% to 6.0%]	0.21
Ankle	13.2% (14/106)	2.4% (68/2795)	**10.9%** [4.4% to 17.3%]	**0.001**
Bilateral pain	48.5% (16/33)	9.3% (259/2795)	**39.6%** [22.5% to 56.7%**]**	** < 0.001**
Chronic pain	51.9% (54/104)	50.8% (706/1389)	**2.0%** [− 8.0% to 12.0%]	0.69
Multisite pain	23.1% (24/104)	16.1% (224/1389)	**7.9%** [− 0.4% to 16.2%]	0.063

The level of significance was set to *p* < 0.05 (in bold)

Confidence intervals (CI) and *p* values from binary linear regression, adjusted for age and sex in total sample. Only those younger than 30 years included

*ADHD* attention-deficit hyperactivity disorder, *CAP* child and adolescent psychiatry, *T3* nine -year follow-up (2018–2019), *HUNT4* the Nord-Trøndelag Health Study 4 (2018–2019)

*Unadjusted for age. Computation did not converge when adjusting for age

In summary, the results indicated significant higher risk for single-site pain in specified locations and multisite pain in those with ADHD, with the highest risk differences in females, as compared to those in the Young-HUNT3 and HUNT4 populations.

## Discussion

This study examined the prevalence, course, and distribution of chronic pain in adolescents with ADHD within a clinical population, along with two age-matched reference population samples. Chronic pain in specified locations and multisite pain were highly prevalent among all adolescents, particularly in females and those with ADHD.

### The prospective course of chronic and multisite pain in those with ADHD

We found that those with ADHD seemed significantly more prone to experiencing single-site and multisite pain at each time of assessment, with females to a greater extent, corresponding well to other studies [28, 33]. ADHD and chronic pain share similar overlapping cognitive and behavioral processes [5, 33, 34]. Neuroinflammation is considered at the origin of ADHD and pain comorbidity [5], but it is unclear how neuroinflammation is related to sex differences in pain prevalence in children. Accordingly, the prevalence of pain in those with ADHD is complex and multifactorial and includes risk factors such as genetics and intergenerational recurrence of ADHD or chronic pain [19, 35]. The prospective decrease in pain prevalence in ADHD patients may be attributable to symptoms in chronic pain states that are episodic and fluctuates in narrow time scale [13, 36]. However, it is evident that previous chronic pain in childhood or adolescents may have strong predictive power for pain later in life [10, 11].

### Difference in pain prevalence

Pain prevalence, including single pain sites, in those with ADHD was significantly higher at all time points as compared to the adolescents and young adults in the general population, and was most prevalent in females.

We found significant risk differences in adolescents with ADHD compared to the general population in reporting single-site pain, particularly in the head, neck, back, abdomen as well as chronic pain and multisite pain. This effect also applied to young adults with ADHD, especially those reporting pain in the neck, back, and lower limbs as well as multisite pain, but not those reporting chronic pain. According to previous studies, experiencing single-site pain does not have substantial impact on everyday functioning; however, the impact increases significantly with increasing numbers of pain sites [11, 12, 28]. In HUNT4, more than half of those with chronic pain reported single-site pain, while 12% of those with chronic pain in CAP-T3 reported single-site pain. Thus, there is a clear need to further understand the clinical relevance of comorbid chronic pain and ADHD in adolescents. Relevant avenues for further research must address the complex sex differences as risk/protective factors concerning the high prevalence of ADHD in males and chronic pain in females [37, 37], also assessing long-term associations with bodyweight, psychiatric comorbidities, and possible mechanisms of stimulant use effects on pain, which were limitations in this study.

The prospective design of this study, acceptable participation rates, and the use of standardized and comparable questions to collect pain data ensured the comparability of results across different studies. We focused on somatic comorbidity in ADHD, which has received less attention in the research literature. In clinical practice, such inattention can lead to misdiagnosis or incorrect treatment, with potentially serious consequences if medical conditions causing the chronic pain are overlooked [39]. However, some limitations need to be considered. The ADHD diagnosis at baseline was based on clinical ICD-10 diagnoses and DSM-IV at T2. Because the criteria for ADHD according to ICD-10 are stricter than the criteria for ADHD in DSM-IV, this may have affected prevalence rates [2]. Even though comorbid psychiatric diagnoses are prevalent in adolescent ADHD, we excluded this perspective. Moreover, the comparison of a clinical population to general reference populations may have produced bias in that the reference populations may have experienced less severe problems, for example more limited impact of pain on functioning as compared to the clinical samples [40]. All samples included in this study are ethnically homogeneous, which limit the generalizability to people of non-European ancestry [18], we relied on self-reporting of pain symptoms rather than clinical findings, suggesting that the complex nature of chronic pain might represent a limitation of this study.

## Conclusion

This longitudinal study of chronic pain in a clinical sample of adolescents and young adults with ADHD compared to the general population found that chronic pain in specified locations as well as multisite pain, was highly prevalent among all adolescents, particularly in females and those with ADHD. To explore the nature of these findings further, we suggest examining the complex sex differences of comorbid chronic pain and ADHD in adolescents in a long-term perspective.

## Data Availability

The dataset used and analyzed during the current study is available from the corresponding author on reasonable request.
